# Molecular characterization of *PRKN* structural variations identified through whole‐genome sequencing

**DOI:** 10.1002/mgg3.482

**Published:** 2018-10-16

**Authors:** Paloma Bravo, Hossein Darvish, Abbas Tafakhori, Luis J. Azcona, Amir Hossein Johari, Faezeh Jamali, Coro Paisán‐Ruiz

**Affiliations:** ^1^ Department of Neurology, Icahn School of Medicine at Mount Sinai One Gustave L. Levy Place New York New York; ^2^ Department of Medical Genetics Semnan University of Medical Sciences Semnan Iran; ^3^ Department of Neurology, School of Medicine, Imam Khomeini Hospital and Iranian Center of Neurological Research Tehran University of Medical Sciences Tehran Iran; ^4^ Department of Neurosciences, Icahn School of Medicine at Mount Sinai One Gustave L. Levy Place New York New York; ^5^ Department of Psychiatry, Icahn School of Medicine at Mount Sinai One Gustave L. Levy Place New York New York; ^6^ Department of Genetics and Genomic sciences, Icahn School of Medicine at Mount Sinai One Gustave L. Levy Place New York New York; ^7^ The Mindich Child Health and Development Institute, Icahn School of Medicine at Mount Sinai One Gustave L. Levy Place New York New York; ^8^ The Friedman Brain and Mindich Child Health and Development Institutes, Icahn School of Medicine at Mount Sinai One Gustave L. Levy Place New York New York

**Keywords:** Parkinson’s disease, *PRKN*, retrotransposition, structural variations, whole‐genome sequencing

## Abstract

**Background:**

Early‐onset Parkinson's disease (PD) is the most common inherited form of parkinsonism, with the *PRKN* gene being the most frequently identified mutated. Exon rearrangements, identified in about 43.2% of the reported PD patients and with higher frequency in specific ethnicities, are the most prevalent *PRKN* mutations reported to date in PD patients.

**Methods:**

In this study, three consanguineous families with early‐onset PD were subjected to whole‐genome sequencing (WGS) analyses that were followed by *Sanger* sequencing and droplet digital PCR to validate and confirm the disease segregation of the identified genomic variations and to determine their parental origin.

**Results:**

Five different *PRKN* structural variations (SVs) were identified. Because the genomic sequences surrounding the break points of the identified SVs might hold important information about their genesis, these were also characterized for the presence of homology and repeated sequences.

**Conclusion:**

We concluded that all identified *PRKN* SVs might originate through retrotransposition events.

## 1 INTRODUCTION

Mutations in the *PRKN* gene (OMIM #600,116) are the most common cause of autosomal recessive Parkinson's disease (PD). The *PRKN* gene is located on chromosome 6q26 and its larger transcript (transcript variant 1; NM_004562.2) contains 12 coding exons and encodes a protein of 465 amino acids (NP_004553.2). All types of mutations, including missense, nonsense, splice site, frameshift, and structural variations (SVs), have been reported in PD patients carrying *PRKN* mutations. SVs (exon rearrangements) are the most common type of mutations, being identified in about 43.2% of the reported patients (Kasten, et al., 2018). In a large multicenter study, where we identified *PRKN* mutations in 71.42% of the examined patients, SVs were the most prevalent mutations identified in the Iranian PD population (Taghavi et al., 2018). The most common *PRKN* SV identified to date in the PD population is the c.(171+1_172‐1)_(412+1_413‐1)del mutation, which consists of a deletion encompassing the entire exon 3 of the *PRKN* gene (https://www.mdsgene.org) (Kasten, et al., 2018). The Multiplex Ligation‐Dependent Probe Amplification (MPLA; MRC Holland), which allows the detection of DNA copy number changes (CNVs) of up to 40 sequences in a single reaction, is the most frequently used technique to identify SVs in the *PRKN* gene, despite the fact that it does not determine the genomic localization of the deletion/insertion break points. On the other hand, whole‐genome sequencing (WGS), considered the most comprehensive genetic screening as it captures both coding and noncoding genetic variation, enables us to identify gene fusions, CNVs, and other complex SVs (Royer‐Bertrand & Rivolta, 2014). The continuous progress of read coverage uniformity and reduced allele bias in WGS (Meynert, Ansari, FitzPatrick, & Taylor, 2014) has led to improved detection of copy number changes and de novo variations (Gilissen, et al., 2014; Ritter, et al., 2015).

## 2 CLINICAL REPORT

We here described the clinical characteristics of three different families carrying *PRKN* SVs. All patients’ clinical details are summarized in Table 1. Briefly, all patients began the disease in childhood, with the youngest patient developing the disease at the age of 10 years. Most of the patients showed slow disease progression with the exception of patient FC‐P2, who onset the disease at the age of 17 and showed severe disease progression (Table 1). Only one family, consisted of three affected siblings, showed additional symptoms (Table 1).

**Table 1:** Clinical details of patients carrying *PRKN* SVs

Patient (gender)	Age at onset	Age	*PRKN*Mutations	Start location	First symptom	Tremor	Progression	Parkinsonism	Rigidity	Postural instability	Dystonia	Bradykinesia	Hypokinesia	Autonomic dysfunction	Pyramidal signs	Stride and sleep apnea	REM sleep behavior disorder	Falling	Response to levodopa	Other symptoms
FA_P1 (Male)	13	62	Delx5/Delx5	Limbs	Resting tremor	Rest, intention, limbs	Slow	Sy	Y	La	Y	Y	Y	Y	*N*	*N*	*N*	Y	Y	Eye lid apraxia or blepharospasm, incontinence, sensory polyneuropathy, impaired smell
FA_P2 (Male)	13	54		Limbs	Resting tremor	Intention, limbs, chin	Slow	Sy	*N*	La	*N*	Y	Y	*N*	*N*	*N*	*N*	Y	Y	Incontinence, sensory polyneuropathy
FA_P3 (Male)	13	50		Whole body	Whole body tremor	Rest, intention, limbs	Slow	Sy	Y	La	*N*	Y	Y	*N*	*N*	*N*	*N*	*N*	Y	Sensory polyneuropathy
FB_P1 (Female)	10	24	Delx2/Delx3	Limbs	Rigidity	Rest	Slow	Asy	Y	Y	Y	Y	*N*	*N*	*N*	*N*	*N*	Y	Y	None
FC_P1 (Female)	17	27	Delx4–6/Delx4	Whole body	Resting tremor	Rest	Fast	Sy	Y	Y	Y	Y	*N*	*N*	*N*	*N*	*N*	Y	*N*	None

Notes

Family A was previously reported in Taghavi et al., 2018 (Family 3).

As: asymmetric; Ea: early; FA_P1: family A, patient 1; La: late; *N*: No; Sy: symmetric; Y: yes.

## 3 METHODS

**Figure 1:**
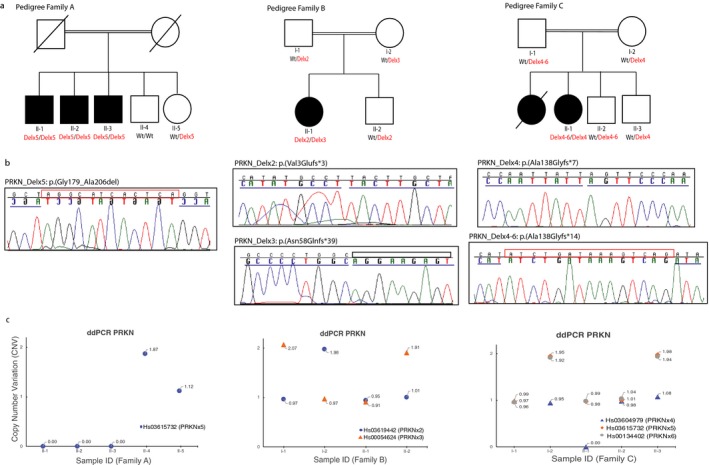
Identification of five different *PRKN* SVs in three families with PD. (a) Pedigree structure of families carrying *PRKN* SVs. Dark squares (males) and circles (females) indicate PD. Wt indicates wild‐type allele. (b) *Sanger* chromatograms of the *PRKN* gene (NM_004562.2) showing the deletions’ break points (highlighted with a blue line). MIs observed in Pedigree A and C are highlighted with a red rectangle. (c) CNVs plots of *PRKN* exons 2, 3, 4, 5, 6 obtained through ddPCR QX100 system, Bio‐rad). Two exon copies (homozygous wt allele) are represented with a CNV score close to 2, one exon copy (heterozygous mutant allele) with a CNV score close to 1, and no copies with a CNV score of 0

Three different families with early‐onset PD were clinically examined and subjected to WGS analyses. The local ethics committee at each participating medical center approved this study, and informed consent, according to the Declaration of Helsinki, was obtained from all participants. DNA samples from all participants were isolated from whole blood, using standard procedures. WGS was performed as previously described (Sanchez, et al., 2016). Specifically, deletions were called by using GenomeSTRiP (v2.0) (Handsaker, Korn, Nemesh, & McCarroll, 2011) and were jointly called by using 17 HapMap individuals (CEPH Platinum Genomes pedigree). All deletions annotated as PASS in the GenomeSTRiP results were further filtered by using custom scripts to remove redundant calls and break points overlapping repeat regions, or with extensive mapping ambiguity. Identified deletions affecting coding areas were further analyzed through SplazerS, which identifies and split‐aligns reads that cross‐structural variant break points (Emde, et al., 2012). First, all reads mapping to the candidate region were extracted, and then by using SplazerS, they were mapped back to the region to identify and confirm the break point locations. Subsequently, *Sanger* sequencing, as described elsewhere (Krebs, et al., 2013) and by using primers flanking the cut‐off points previously determined by the WGS analyses, was used to validate the identified deletions and to determine the genomic localization of the deletions’ break points. Primer sequences were designed by using a public primer design website (https://ihg.gsf.de/ihg/ExonPrimer.html; primer sequences available upon request) using the NM_004562.2 gene sequence as a reference. The validated CNVs were later quantified through the ddPCR QX100 system (Bio‐rad, USA) by using TaqMan probes targeting *PRKN* exons 2–6 as well as a reference gene (TERT) (Hindson, et al., 2011). Taqman probes were acquired from Applied Biosystems (Life Technologies, USA), and a DNA sample from a healthy individual as well as a non‐template control were, respectively, used as reference control DNA and negative control. All CNV scores were calculated using the Quantasoft software according to the manufacturer's instructions (Bio‐Rad, USA). These analyses were done in all available family members in order to examine the disease segregation of the deletions and to determine their parental origin (Figure 1).

The formation of SV is a complex phenomenon that is not well understood. Long homologies around break point suggest SV formation by nonallelic homologous recombination (NAHR); short homologies, with high mobile element content within SV regions, indicate that they originated through transposable element insertions (TEI); while little or no homology suggests SV formation by a nonhomologous end‐joining (NHEJ) or by a template‐switching mechanism during replication. We therefore examined the proximal and distal sequences (~2 kb) to the break points to determine their homology and the presence of repeated sequences, as they are known to affect the genomic integrity through recombination involving insertion, deletion, and rearrangements (Abyzov, et al., 2015; Kaer & Speek, 2013; Lupski & Stankiewicz, 2005). First, pairwise sequence comparisons were carried out to determine the homology between the sequences flanking the deletions’ break points. Both proximal and distal sequences to the deletions’ break points were aligned through Clustal Omega software (https://www.ebi.ac.uk/Tools/msa/clustalo/). Data from the NIH Roadmap Epigenomics project (https://www.roadmapepigenomics.org/) were used to examine the deletions’ break points for the presence of chromatin marks and repeated elements, such as retrotransposons, including the long terminal repeat (LTR) and the non‐LTR retrotransposons (i.e.; long interspersed elements (LINEs or L1) and short interspersed element (SINEs)) (Kaer & Speek, 2013). Data for the chromatin states using a multivariate Hidden Markov Model (HMM; ChromHMM analysis) were also investigated (Ernst, et al., 2011).

## 4 RESULTS AND DISCUSSION

We identified five different PRKN deletions. Family A was previously reported and shown to carry a homozygous PRKN exon 5 deletion (NG_008289.2: g.574615_702745del) that was identified through WGS (Taghavi et al., 2018). Here, we described the identified mutation (c.534+42943_618+24033delinsAGGCATCACTCA) and its effect on the protein (p.(Gly179_Ala206del)) following the guidelines of the Human Genome Variation Society (https://www.hgvs.org/) and the Mutalyzer program (https://www.LOVD.nl/mutalyzer/) (Figure 1, Table 2) (https://databases.lovd.nl/shared/variants/0,000,368,892).

**Table 2:** *PRKN* deletions identified through whole‐genome sequencing in three different PD families

Family	Chr	Break points		Mutations (NG_008289.2 (*PRKN*_ NM_004562.2)		Implicated exons	Size (bp)
		Position (5′)	Position (3′)	Nucleotide change	Protein change		
Ped. A	6	162,451,090	162,579,220	c.534+42943_618+24033delinsAGGCATCACTCA	p.(Gly179_Ala206del)	Exon 5	128,130
Ped. B	6	162,837,521	162,925,282	c.8‐60777_171+26821del	p.(Val3Glufs*3)	Exon 2	87,761
	6	162,655,261	162,724,846	c.172‐41049_412+28296del	p.(Asn58Glnfs*39)	Exon 3	69,585
Ped. C	6	162,605,414	162,641,899	c.413‐19615_534+16749del	p.(Ala138Glyfs*7)	Exon 4	36,485
	6	162,340,003	162,667,521	c.412+16037_734+54332delinsATCTGATAAAGTCAG	p.(Ala138Glyfs*14)	Exons 4, 5, 6	327,518

Additionally, we identified two isolated PD cases carrying compound heterozygous *PRKN* deletions and characterized all five *PRKN* deletions. In the new cases, the performed WGS analyses led to the identification of 683 and 592 coding (including missense, nonsense, and frameshift) and splice site nucleotide variations for patient B_II‐1 and patient C_II‐1, respectively. Because both patients were born to consanguineous marriages, a recessive pattern of inheritance was suspected (Figure 1). However, no rare (with a frequency <0.5% for a recessive model) or novel homozygous or compound heterozygous coding variations were identified in the patients’ genomes, meaning that all variations identified were present in heterozygosis and therefore were not compatible with a recessive pattern of inheritance. All known coding variations (known and unknown) identified in the known PD genes were as well examined, but no pathogenic mutation was identified. We then also examined all SVs identified through WGS in the patients’ genomes. Patient B_II‐1 was shown to carry 50 SVs while 56 different SVs were identified in the patient C_II‐1. Interestingly, we found that both patients carried two different heterozygous SNVs at the *PRKN* locus. The PD patient from Family B was shown to carry heterozygous* PRKN* deletions involving exon 2 (NG_008289.2:g.228553_316314del; https://databases.lovd.nl/shared/variants/0,000,368,960) and exon 3 (NG_008289.2:g.428989_498574del; https://databases.lovd.nl/shared/variants/0,000,368,961), respectively, while the patient from Family C was shown to carry a heterozygous *PRKN* exon 4 deletion (NG_008289.2:g.511936_548421del; https://databases.lovd.nl/shared/variants/0000368999) and another heterozygous deletion involving *PRKN* exons 4–6 (NG_008289.2:g.486315_813833del; https://databases.lovd.nl/shared/variants/0000368998) (Table 2; Figure 1b,c). In addition, we identified through *Sanger* sequencing small micro‐insertions (MIs) of 12 and 15 bp, respectively, in the patients carrying the two largest deletions (~128 and ~327 kb; Figure 1b, Table 2). All deletions segregated with disease status as only the patients were carriers of two mutant alleles, as expected for an autosomal recessive inheritance (Figure 1a–c; Table 2). The nomenclature of each identified deletion was checked in the Mutalyzer program, with which we examined their effect on the protein. All deletions but one were predicted to cause premature stop codons (Table 2), thus resulting in truncated, nonfunctional proteins.

We found long terminal repeat (LTR) and non‐LTR retrotransposons at both proximal and distal break point regions along with short DNA homologies around the deletions’ break points (the majority of them close to the deletions’ break points or <1 kb away [Tables 2 and 3]), indicating that all these deletions might originate through TEI (Lupski & Stankiewicz, 2005). All proximal and distal regions showed repressive marks, such as tri‐methylation at H3K9 and H3K27 (H3K9me3, H3K27me3). Although the majority of genomic regions surrounding the deletions’ break points also showed methylation and/or tri‐methylation at H3K4 (H3K4me3, H3K4me1), which influences transcriptional activation, depletion of active marks was observed in some of the break points, as it has been observed in other TEI break points (Abyzov, et al., 2015) (Table 3).

**Table 3:** Retrotransposons identified within 1–2 Kb upstream and downstream regions to the deletions’ break points

Fam	Mutation (NG_008289.2)	Upstream Repeats				Downstream Repeats				Mechanisms
		Position	Size (bp)	Type	Chromatin marks	Position	Size (bp)	Type	Chromatin marks	
Ped. A	g.574615_702745del	162,450,885–162,451,171	286	SINE (AluJr4)	H3K9me3 H3K27me3	162,579,449–162,579,761	312	SINE (AluSz)	H3K4me1 H3K4me3 H3K9me3 H3K27me3	TEI
Ped. B	g.228553_316314del	162,836,570–162,836,852	282	SINE (AluSz)	H3K4me1 H3K4me3 H3K9me3 H3K27me3	162,925,457–162,926,411	954	LINE (L1ME3C)	H3K4me1 H3K4me3 H3K9me3 H3K27me3	TEI
	g.428989_498574del	162,654,701–162,655,043	342	LTR (MSTD)	H3K4me1 H3K4me3 H3K9me3 H3K27me3	162,724,757–162,725,149	392	LINE (L1MC4A)	H3K9me3 H3K27me3	TEI
Ped. C	g.511936_548421del	162,605,380–162,605,745	365	LTR (THE1C)	H3K4me1 H3K4me3 H3K9me3 H3K27me3	162,643,141–162,643,834	693	LTR (MLT1E1A)	H3K4me1 H3K4me3 H3K9me3 H3K27me3	TEI
	g.486315_813833del	162,339,759–162,340,554	795	LINE (L1ME1)	H3K4me1 H3K4me3 H3K9me3 H3K27me3	162,669,915–162,670,199	284	SINE (AluSc)	H3K4me1 H3K9me3 K27me3	TEI

We concluded that WGS is the preferred technique to well characterize the copy number changes observed in the* PRKN* gene as well as other parkinsonism genes, as it enables us to characterize the bases around their break points that are thought to hold important information about their genesis (Kidd, et al., 2010). Because exon rearrangements are the most common mutations identified in the *PRKN* gene and in recessive PD, since the *PRKN* gene is the most frequently mutated gene, the characterization of *PRKN* SVs is essential for understanding their formation mechanisms as well as for examining and interpreting their functional effects in model organisms. Taken together, precise mapping of deletion break points and localization of the repeated elements is important because they might reveal common disease signatures that will, in turn, lead to novel genomic editing strategies for gene therapy (Esposito, et al., 2017; Kaer & Speek, 2013; Lupski & Stankiewicz, 2005).

## DISCLOSURE STATEMENT

The authors declare no conflict of interest.
